# Genome-Wide DNA Methylation Profiles in Community Members Exposed to the World Trade Center Disaster

**DOI:** 10.3390/ijerph17155493

**Published:** 2020-07-30

**Authors:** Alan A. Arslan, Stephanie Tuminello, Lei Yang, Yian Zhang, Nedim Durmus, Matija Snuderl, Adriana Heguy, Anne Zeleniuch-Jacquotte, Yongzhao Shao, Joan Reibman

**Affiliations:** 1 Department of Obstetrics and Gynecology, New York University Langone Health, New York, NY 10016, USA; 2 Department of Population Health, New York University Langone Health, New York, NY 10016, USA; Stephanie.Tuminello@nyulangone.org (S.T.); ly888@nyu.edu (L.Y.); Yian.Zhang@nyulangone.org (Y.Z.); Anne.Jacquotte@nyulangone.org (A.Z.-J.); Yongzhao.Shao@nyulangone.org (Y.S.); 3 NYU Perlmutter Comprehensive Cancer Center, New York, NY 10016, USA; 4 Department of Medicine, New York University Langone Health, New York, NY 10016, USA; Nedim.Durmus@nyulangone.org (N.D.); Joan.Reibman@nyulangone.org (J.R.); 5 Department of Pathology, New York University Langone Health, New York, NY 10016, USA; Matija.Snuderl@nyulangone.org (M.S.); Adriana.Heguy@nyulangone.org (A.H.); 6 NYU Langone’s Genome Technology Center, New York, NY 10016, USA

**Keywords:** environmental exposure, epigenome-wide association study, exposure assessment, methylation, pathway analysis, World Trade Center, 9/11

## Abstract

The primary goal of this pilot study was to assess feasibility of studies among local community members to address the hypothesis that complex exposures to the World Trade Center (WTC) dust and fumes resulted in long-term epigenetic changes. We enrolled 18 WTC-exposed cancer-free women from the WTC Environmental Health Center (WTC EHC) who agreed to donate blood samples during their standard clinical visits. As a reference WTC unexposed group, we randomly selected 24 age-matched cancer-free women from an existing prospective cohort who donated blood samples before 11 September 2001. The global DNA methylation analyses were performed using Illumina Infinium MethylationEpic arrays. Statistical analyses were performed using R Bioconductor package. Functional genomic analyses were done by mapping the top 5000 differentially expressed CpG sites to the Kyoto Encyclopedia of Genes and Genomes (KEGG) Pathway database. Among cancer-free subjects, we observed substantial methylation differences between WTC-exposed and unexposed women. The top 15 differentially methylated gene probes included BCAS2, OSGIN1, BMI1, EEF1A2, SPTBN5, CHD8, CDCA7L, AIDA, DDN, SNORD45C, ZFAND6, ARHGEF7, UBXN8, USF1, and USP12. Several cancer-related pathways were enriched in the WTC-exposed subjects, including endocytosis, mitogen-activated protein kinase (MAPK), viral carcinogenesis, as well as Ras-associated protein-1 (Rap1) and mammalian target of rapamycin (mTOR) signaling. The study provides preliminary data on substantial differences in DNA methylation between WTC-exposed and unexposed populations that require validation in further studies.

## 1. Introduction

The collapse of the World Trade Center (WTC) towers on September 11th, 2001 was an unprecedented disaster, with rescue workers, local workers, residents, and commuters exposed to the dust and smoke from the buildings’ pulverization and subsequent fires [1]. WTC dust was comprised mostly of building materials such as cement, cellulose, and glass fibers, but also contained asbestos [1]. Incomplete combustion and unburnt jet fuel released polycyclic hydrocarbons and phthalates [2]. Also measured in the settled dust/smoke were polychlorinated chlorinated biphenols (PCBs), polybrominated diphenol ethers (PBDEs), dioxins, furans, and polycyclic aromatic hydrocarbons (PAHs), as well as heavy metals including arsenic, beryllium, cadmium, chromium, nickel, and other elements such as copper, lead, and mercury [2,3,4,5].

Environmental exposures, including metals, benzene, polycyclic aromatic hydrocarbons, and organic pollutants, can all induce epigenetic changes [6,7]. Epigenetic modification occurs when hereditable changes are made that alter gene expression without changing the underlying genetic sequence [7,8]. CG dinucleotides (CpGs) sites are platforms for DNA methylation, a significant epigenetic mechanism of the human genome [8]. Generally, CpG methylation induces gene silencing through inhibition of transcription factor binding to the promotor regions of genes, inhibition of RNA polymerase, and recruitment of transcriptional repressor complexes [8]. Aberrant DNA methylation has been implicated in multiple human diseases, including cancer [7,9].

Methylation marks can have clinical utility. For example, Morrow et al. recently showed that DNA taken from peripheral blood of smokers could be used to identify methylation sites predictive of mortality [10]. However, despite the complex assortment of WTC-related environmental exposures, and the poor health outcomes related to that exposure [11,12], the epigenetic consequences of the WTC disaster have been largely unexplored. Looking at the DNA methylation profile of WTC responders according to an exposure index, Kuan et al. has recently reported preliminary evidence of a methylation signature for cancer-related exposure in this population [13]. However, the epigenetic impact of WTC exposure in residents and local workers (also known as “survivors”) has never been examined.

Populations not involved in rescue and recovery activities (community members) remain understudied in terms of WTC exposures and related outcomes. These populations include those who were working in the WTC towers or in the many surrounding offices, stores, and restaurants (local workers) as well as residents of the surrounding buildings (residents). Over 36,000 local workers and over 57,000 residents south of Canal Street in lower Manhattan alone have been estimated to have had potential for dust and fume exposure [14]. Additional work-exposed populations include those involved in the cleanup of the surrounding area (cleanup workers). For some community members, exposure to the dust and smoke persisted for weeks or months after the event, with many cleanup workers not wearing the proper respiratory protection, and many residents returning to inadequately cleaned buildings [2].

We hypothesized that high-dose and prolonged WTC dust and fume exposures may have created persistent epigenetic changes in the community members exposed to the WTC disaster. We performed a pilot study to assess the feasibility of blood DNA collection among community members from the Bellevue Hospital WTC Environmental Health Center (WTC EHC) cohort, with the objective of comparing their genome-wide DNA methylation profile to a reference group of New York City residents from the NYU Women’s Health Study (NYUWHS) prospective cohort, who donated blood samples before 9 December 2001. As a secondary objective we aimed to characterize these changes, specifically in terms of gene expression pathways that may have been altered.

## 2. Methods

### 2.1. Study Participants

#### 2.1.1. WTC Environmental Health Center (WTC EHC)

As part of the World Trade Center Health Program (WTCHP), created by the Centers for Disease Control (CDC) and the National Institute of Safety and Occupational Health (NIOSH), the Bellevue Hospital WTC EHC is the only program that provides medical and mental health treatment for local community members and cleanup workers exposed to the WTC disaster [15,16]. The WTC EHC program started through joint efforts of the local community, organized labor, and the medical community [15]. This program became law (James Zadroga 9/11 Health and Compensation Act H.R. 847) in 2010 for implementation in 2011. Conditions which could be included in this program were deemed “certifiable illnesses” and included pulmonary, digestive, and mental health disorders. Cancer was added as a “certifiable” condition in 2012.

In contrast to the responder programs, the WTC EHC includes a substantial women population (~50%) and is ethnically diverse. Community members self-refer into this program and under law inclusion requires the presence of a “certifiable condition” linked to the WTC disaster [16]. As of 31 December 2019, 11,048 individuals have been enrolled in the WTC EHC program, with 2840 individuals diagnosed with cancer. Between March and September 2018, we enrolled and collected samples from 18 WTC-exposed cancer-free women from the WTC EHC.

#### 2.1.2. New York University Women’s Health Study (NYUWHS)

The reference group of subjects was selected from the New York City women who donated blood samples before 9 December 2001 as part of the NYUWHS prospective cohort. Between March 1985 and June 1991, 14,274 women between the ages of 35 and 65 years were enrolled as volunteers in the NYUWHS at the Guttman Breast Diagnostic Institute, a mammography screening center in New York City. Eligibility was restricted to women who had not used hormonal medications, or been pregnant or lactating, in the preceding 6 months. Subjects completed a self-administered baseline questionnaire that collected information on demographic, medical, reproductive, regular physical activity, and anthropometric variables, as well as recent medication use. Cohort participation required donation of venous blood, drawn using collection tubes with and without anticoagulant. With rare exceptions, blood collection took place between 9:30 AM and 1:00 PM. Subjects were not asked to fast but time of last meal was recorded. Exact time at venipuncture was also recorded. After drawing, tubes were kept covered at room temperature (25 °C) for 15 min, then at 4 °C for 60 min to allow clot retraction and then centrifuged. Supernatant serum was partitioned into 1 mL aliquots in capped plastic vials within 2 h after separation. Up to 12 labeled aliquots per blood sample were immediately banked at −80 °C. Beginning in 1988, two 1 mL aliquots of the cellular precipitate from centrifuging the blood were stored at −80 °C. Beginning in 1989, blood clots were also collected and stored at −40 °C as an additional source of DNA in sealable plastic-lined aluminum bags.

The NYUWHS provided de-identified cellular precipitate samples of 24 age-matched cancer-free women as a reference group for the current study.

### 2.2. Blood Sample Collection, Nucleic Acid Isolation, and DNA Processing

After obtaining informed consent, blood samples from 18 cancer-free WTC EHC women were collected during their routine clinical visit to the WTC EHC and immediately centrifuged and processed to separate white blood cells (buffy coat) using standard protocol [17]. Reference samples from 24 age-matched cancer-free NYUWHS women were identified and retrieved from storage. Both WTC EHC and NYUWHS buffy coat samples were sent to the NYU Langone’s Biospecimen Research and Development (CBRD) laboratory for DNA extraction. DNA was recovered using the PicoPure DNA extraction kit (Thermo Fisher Scientific, Boston, MA, USA), which enables recovery of genomic DNA from formalin-fixed, paraffin-embedded tissue. DNA was then subjected to bead purification with the Sphere quality control kit (Thermo Fisher Scientific, Boston, MA, USA). DNA purity and quantity were assessed using NanoDrop spectrophotometer (NanoDrop Technologies, Wilmington, DE, USA). The DNA was bisulfite converted using the EZ-96 DNA methylation kit (Zymo Research, Irvine, CA, USA). Extracted DNA was restored using the Infinium HD formalin-fixed, paraffin-embedded DNA restore kit (Illumina^®^, San Diego, CA, USA) prior to hybridization on the bead chips provided by the manufacturer (Illumina^®^).

### 2.3. Differential Methylation Analysis and Unsupervised Hierarchical Clustering

The Infinium Methylation EPIC array (Illumina^®^) was used to determine the DNA methylation status of 866,562 CpG sites, following the manufacturer’s instructions. All statistical analysis and modeling were performed using the open-source software *R*. Minfi *R* package was used to process and analyze the methylation data [18]. Using minfi package, the probes were quantile normalized and background adjusted. The resulting set of samples and probes was used for differentially methylated probes analysis. The differentially methylated probes Finder function in Minfi package was used to identify differentially methylated probes between WTC EHC (exposed) and NYUWHS (unexposed) samples. Probes with statistical significance using Benjamini–Hochberg false discovery rate *q* cutoff of *q* < 0.05 were considered most significant, and corresponding heatmap is shown. *β*-values for all 866,562 CpG sites tested were defined as the ratio of fluorescence intensity of the methylated probe over the overall intensity of probes. *β*  <  0.2 indicated hypomethylation (blue); *β*  >  0.8 indicated hypermethylation (red). All graphs and heatmaps were generated using the *R* package. Unsupervised hierarchical clustering was done with Euclidean measure for distance matrix and complete agglomeration method for clustering was used for unsupervised hierarchical clustering.

### 2.4. Functional Genomic Pathway Analysis

All probes included in the array were annotated using the HumanMethylation850 manifest provided by the manufacturer (MethylationEPIC_v-1-0_B4; Illumina, San Diego, CA, USA). Genomic information, including DNA sequence and coordinates of gene-coding regions, were obtained from the University of California Santa Cruz Genome Browser database [19]. All probes covering promoters and enhancers of coding genes were filtered in and considered for the enrichment pathway network analysis. This rationale was adopted to limit nonspecific enrichment pathway results that may occur when all coding and noncoding genes are included. Using the R package, we ran in parallel Cluster profiler and the DAVID Bioinformatics Resources interrogating 5867 genes in order to detect differentially methylated genes [20,21,22]. The enrichment test is based on the Fisher exact test, which indicates if the overlap between genes in a cluster and in a Gene Ontology term is higher than expected by chance. All sources of interaction evidence are calibrated against previous knowledge using the high-level functional groupings provided by the Kyoto Encyclopedia of Genes and Genomes (KEGG) pathway map [23].

## 3. Results

Basic descriptive characteristics of WTC-exposed (WTC EHC) and unexposed (NYUWHS) women are presented in Table 1. The comparison groups were comparable in terms of age at blood donation. Compared to the WTC EHC women, NYUWHS subjects had a higher proportion of Caucasian (44.4% and 79.2%, respectively) and lower proportion of Hispanic (44.4% vs. 12.5%, respectively) women, reflecting the ethnic distributions in respective study sites. Among the WTC EHC subjects, 12 subjects reported acute WTC dust cloud exposure on 11 September 2001 and six subjects reported exposure to the WTC re-suspended dust and fumes between 11 September 2001 and 31 December 2001, reflecting chronic exposure.

Focusing on the top 5000 differentially expressed probes between WTC-exposed and unexposed women, we observed that the majority of probes (4778 out of 5000; 95.6%) had higher mean methylation values among the WTC-exposed women compared to unexposed women. Only 222 (4.4%) out of the top 5000 differentially methylated probes had a mean methylation value lower in the WTC EHC compared to the NYUWHS participants.

**Figure 1:**
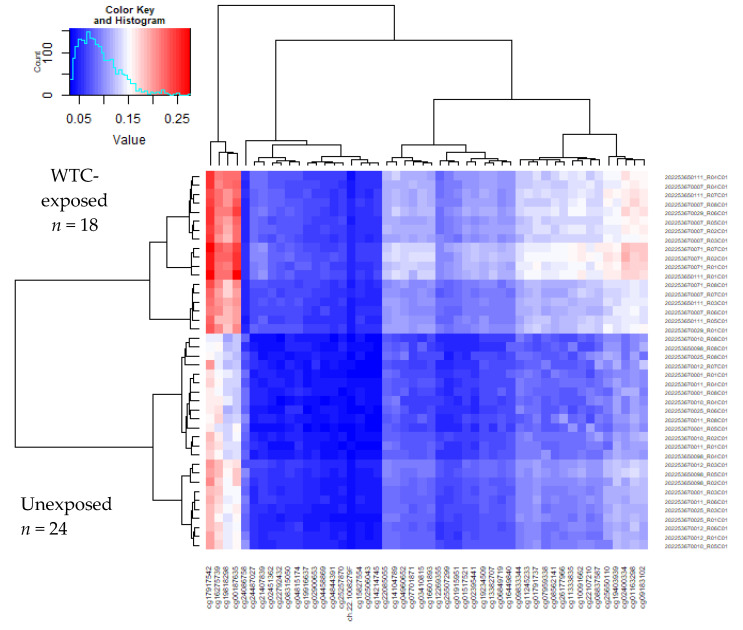
Unsupervised hierarchial clustering heatmap for the 50 most significantly differentially methylated CpG sites between the WTC-exposed (WTC EHC) vs. unexposed (NYUWHS) cancer-free subjects.

Figure 1 presents the heatmap based on the top 50 differentially methylated probe sets. Unsupervised hierarchical clustering focusing on the top 50 differentially methylated probes showed different patterns of methylation among WTC-exposed and unexposed women.

**Figure 2:**
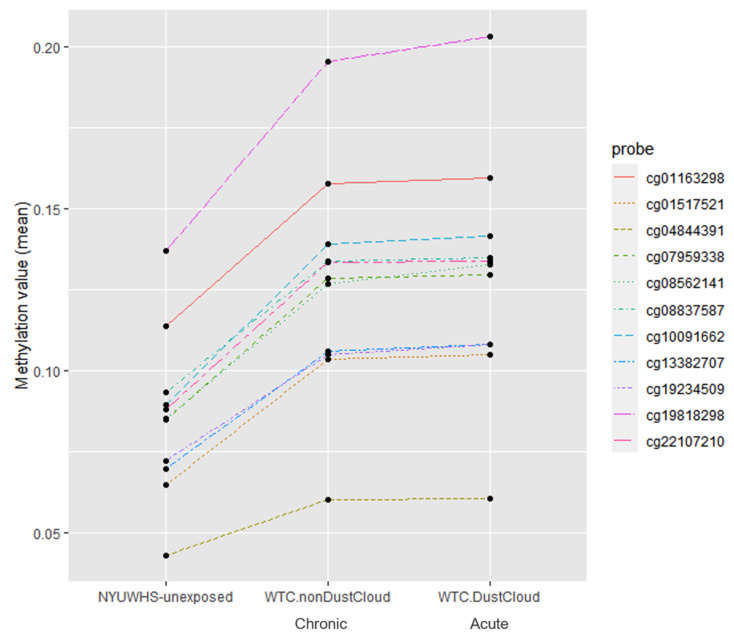
Mean methylation values of the top 11 differentially methylated probes of the WTC exposed groups (no dust cloud and dust cloud exposure) compared to the unexposed group (NYUWHS).

Table 2 lists the top 15 differentially methylated probe sets between WTC-exposed and unexposed women. All 15 CpG sites had higher mean methylation values in the WTC exposed community members compared to the unexposed NYUWHS group (Table 2). Eleven out of the top 15 differentially methylated sites had higher mean methylation values in both WTC EHC subgroups: acutely exposed to the WTC dust cloud on 11 September 2001 and chronically exposed to the WTC dust after 11 September 2001 compared to WTC unexposed subjects (Figure 2). The top two differentially methylated CpG sites were cg19234509 and cg00187635, associated with genes BCAS2 and OSGIN1, respectively.

We have compared the methylation status of established tumor suppressor genes and oncogenes [24] between the WTC exposed and unexposed subjects. Sixteen out of 24 tumor suppressor genes had higher mean methylation values in the promoter region of the WTC exposed subjects compared to unexposed subjects (Table 3). Similarly, compared to the unexposed subjects, WTC exposed subjects had higher mean methylation values at the locations of known oncogenes (Supplementary Table S1).

**Figure 3:**
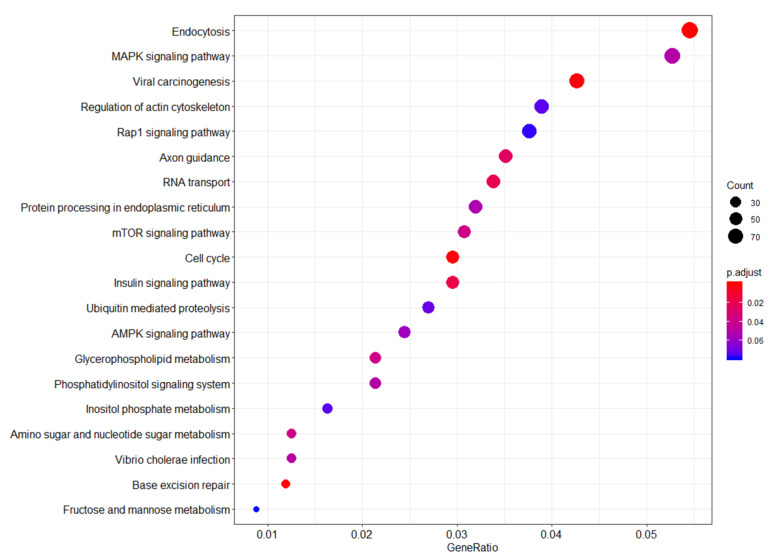
Functional genomic pathways enriched in the WTC-exposed (WTC EHC) cancer-free subjects compared to the unexposed cancer-free subjects (NYUWHS). Summary of pathway network analysis highlights the relationship between probe sets enriched in the WTC-exposed women compared to unexposed women. *Y*-axis shows the probe sets with significant overlap with the reference probe sets/genes from the KEGG database. *X*-axis shows the ratio of the number of differentially expressed probe sets/genes to the total number of genes included in the particular pathway gene set from the reference KEGG pathway database. The dot sizes are proportional to the number of overlapping probe sets/genes. The dot colors show the p-value adjusted for false discovery rate.

Functional gene pathway enrichment analysis revealed that genes in the endocytosis, mitogen-activated protein kinase (MAPK) signaling, and viral carcinogenesis pathways were enriched in the WTC EHC community members compared to the NYUWHS controls (Figure 3). For all of these biological pathways the ratio of observed differentially methylated genes out of the total number of genes in the corresponding KEGG pathway reference set is between 4% and 5%. Among the top 15 functional pathways up-regulated in the WTC-exposed women, MAPK, Ras-associated protein-1 (RAP1), mammalian target of rapamycin (mTOR), and the AMP-activated protein kinase (AMPK) signaling pathways and the phosphatidylinositol signaling system are all involved in signal transduction for environmental information processing [25]. RNA transport, protein processing in the endoplasmic reticulum, ubiquitin mediated proteolysis and base excision repair are all vital pathways for genetic information processing [25].

## 4. Discussion

The present study is one of the first to directly compare genome-wide DNA methylation profiles of WTC exposed (WTC EHC) vs. WTC unexposed (NYUWHS) individuals without cancer. This is also the first analysis to investigate the epigenetic profiles of the WTC-exposed community members who were free of cancer at the time of blood donation. The study demonstrates the feasibility of obtaining blood samples from the WTC-exposed community members, collected as part of routine clinical care, for comprehensive DNA methylation analysis. Given the size and patient diversity of the WTC EHC program, this provides an opportunity for the testing of genetic and epigenetic consequences of the WTC exposures among local community members.

Moreover, our preliminary findings could have important implications for more objective assessment of WTC exposures, and should be more thoroughly explored in future studies of local community and respondent populations. Unsupervised cluster analysis showed distinct patterns of methylation based on the WTC exposure status suggesting that methylation changes related to the WTC exposures are persistent and can be detected many years after the original exposure. The top two differentially methylated CpG sites cg00187635 (BCAS2) and cg19234509 (OSGIN1) have important biological implications. Breast cancer amplified sequence 2 (BCAS2) is located on chromosome 1 and is thought as having multiple roles in development of breast cancer, possibly through its interaction with estrogen receptor alpha [26], or as a negative regulator of the p53 tumor suppressor gene [27]. Oxidative stress induced growth inhibitor 1(OSGIN1) is a tumor suppressor gene linked to cellular stress and apoptosis, variants of which have been implicated in cancer development, specifically in hepatocellular carcinoma [28].

Among the rest of the top 15 differentially methylated probes, there were several other genes that may play a role in cancer development. BMI1 is a cancer stem cell marker [29] that has been implicated in acute myeloid leukemia [30], cervical cancer [31], and prostate cancer [32], among others, by acting as a chromatic remodeler of regulatory genes. EEF1A2 expression is elevated in poor prognosis of triple negative breast cancers [33]. CDCA7L is important for cell cycle regulation and is dysregulated in multiple cancers [34]. ARHGEF7 plays a role in cytoskeleton remodeling, and may regulate cancer cell motility [35], USF1 expression is related to cellular stress and senescence, immune response and carcinogenesis and has been shown to be associated with shorter life expectancy [36]. USP12 is a deubiquitinating enzyme that plays a critical role in TP53 tumor suppressor levels [37].

Functional genomic pathway analysis revealed in the WTC-exposed local community group upregulation of several cancer-related pathways, including the MAPK signaling pathway, viral carcinogenesis, Rap1 signaling pathway, cell cycle, ubiquitin-mediated proteolysis, AMPK signaling pathway, phosphatidylinositol signaling system, amino sugar and nucleotide sugar metabolism, and base excision repair. A recent analysis by Morrow et al. examined sites of DNA methylation predictive of mortality among smokers identified seven CpG predicate sites [10], two of which (corresponding to genes FAM178B and USP2) were found to have higher mean methylation values among WTC-exposed local community members in our study. FAM178B’s function is understudied, but it may play a role in the CD34+ hematopoietic stem lineage [38]. USP2 is a ubiquitin-specific protease found to be capable of promoting metastasis in triple negative breast cancer [39]. While all participants in our study were cancer-free, an increase in cancer incidence is well documented among WTC-exposed individuals. Three separate cohort studies have shown overall cancer rates of those exposed to WTC dust are 6–14% higher than expected [11,40,41].

We observed that the majority of probes had higher mean methylation values among the WTC-exposed women (including both groups: acutely exposed to the dust cloud on 11 September 2001 and chronically exposed to re-suspended WTC dust after 11 September 2001) compared to unexposed women in the current study. Hypermethylation of the promoter region is usually associated with gene silencing and is a key event in carcinogenesis [42]. There were several tumor suppressor genes implicated in cancer susceptibility, including both high penetrance genes (i.e., NF1, PTEN) [43,44,45] and lower penetrance genes (PALB2, ATM) [46,47,48], that were found to have higher mean methylation values in the promoter regions of WTC exposed compared to unexposed individuals. Our preliminary results suggest an epigenetic link between the WTC exposure and cancer, although this potential carcinogenic mechanism requires further validation. WTC-exposure related health effects are not, however, limited to cancer, and other potential epigenetic-mediated impacts also warrant consideration.

This work is preliminary feasibility study, and as such there are limitations that must be acknowledged. Our sample size was small and limited to women only, so we cannot comment here on the DNA methylation profiles of WTC-exposed male community members. There is also the possibility of selection bias as the WTC-exposed community members enrolled into our study were women who sought out regular WTC EHC clinical care instead of being randomly selected from the entire WTC EHC cohort. Moreover, blood was collected from local community members more than 15 years after the WTC disaster, and while DNA methylation changes are known to be persistent [8], it is unknown how long epigenetic changes that occurred as a consequence of WTC-exposure might last. DNA for methylation analysis was also collected from mixed white blood cells, and blood cell type may impact DNA methylation profiles [49]. Given the small sample size, it was challenging to perform subgroup analyses and take into account potential confounders. We are collecting detailed documentation of psychological exposures and relevant mental health symptoms (PTSD, anxiety, depression), lung function abnormalities, neuropathic symptoms and other comorbidities, as well as income information in the WTC EHC and NYUWHS cohorts and will account for these and other potential confounders in the future larger study of these cohorts. Lastly, we were unable to quantify the level of WTC exposure of the local community members. However, we saw an overlap of multiple differentially methylated genes that Kuan et al. found to be related to increased WTC exposure status, including NOTCH4, GRIN2A, IFNAR2, and MPP5, among others [13].

Our study had several strengths including the availability of the control group of randomly chosen individuals without WTC exposure with blood samples collected prior to 11 September 2001. We used the latest global methylation platform from Illumina to assess methylation status at more than 850,000 methylation sites. This was also the first study to look at DNA methylation in the WTC EHC local community members, and as such we included an ethnically diverse group of women with WTC exposures, a group that has traditionally been understudied in WTC-related research.

## 5. Conclusions

In conclusion, we demonstrated feasibility of performing comprehensive genetic and epigenetic studies in the WTC EHC, with preliminary data that WTC-exposure manifests as DNA methylation changes evident many years after the initial WTC exposure. Several of the differentially-methylated genes between the WTC-exposed and unexposed women were previously described to play a role in cancer. As those exposed to the WTC disaster continue to age and adverse health issues become increasingly more common, understanding the epigenetic mechanisms behind these conditions will become even more important, with implications for exposure assessment and health effects evaluation. These results, however, require further replication and validation by larger studies, including both local community members as well as first respondents.

## Figures and Tables

**Table 1:** Descriptive characteristics of the WTC-exposed (WTC EHC) and unexposed (NYUWHS) cancer-free subjects.

Characteristic	WTC EHC *n* = 18	NYUWHS *n* = 24
Age at blood donation, mean (SD)	57.4 (8.4)	56.0 (8.3)
Race/ethnicity, *n* (%)		
Caucasian	8 (44.4%)	19 (79.2%)
Hispanic	8 (44.4%)	3 (12.5%)
African-American	1 (5.6%)	2 (8.3%)
Asian	1 (5.6%)	0 (0%)
Body mass index, *n* (%)		
Normal weight (<25)	7 (39%)	13 (54%)
Overweight (25–30)	6 (33%)	6 (25%)
Obese (≥30)	5 (28%)	5 (21%)
Smoking, (*n*%)		
Never	12 (67%)	12 (50%)
Former	5 (28%)	10 (42%)
Current	1 (5%)	2 (8%)
Location status, *n* (%)		
Resident	5 (27%)	24 (100%)
Local worker	11 (61%)	
Clean-up worker	2 (11%)	
WTC dust exposure, *n* (%)		
Acute, on 9/11	12 (67%)	-
Chronic, post 9/11	6 (33%)	

**Table 2:** Top 15 differentially methylated probes of the WTC-exposed (WTC EHC) vs. unexposed (NYUWHS) cancer-free subjects.

Gene(s)	Probe ID	WTC EHC Methylation Value, Mean	NYUWHS Methylation Value, Mean	*p* Value	*q* Value
BCAS2	cg19234509	0.107	0.072	6.29 × 10^−17^	1.09 × 10^−11^
OSGIN1	cg00187635	0.224	0.145	1.67 × 10^−16^	1.09 × 10^−11^
BMI1	cg10091662	0.141	0.089	1.76 × 10^−16^	1.09 × 10^−11^
EEF1A2	cg01915951	0.096	0.060	2.06 × 10^−16^	1.09 × 10^−11^
SPTBN5	cg25507299	0.092	0.058	2.20 × 10^−16^	1.09 × 10^−11^
CHD8	cg19818298	0.201	0.137	2.49 × 10^−16^	1.09 × 10^−11^
CDCA7L	cg22107210	0.134	0.088	2.63 × 10^−16^	1.09 × 10^−11^
AIDA (C1orf58)	cg04844391	0.061	0.043	2.98 × 10^−16^	1.09 × 10^−11^
DDN	cg01163298	0.159	0.114	3.15 × 10^−16^	1.09 × 10^−11^
SNORD45C (RABGGTB)	cg11245233	0.130	0.090	3.53 × 10^−16^	1.10 × 10^−11^
ZFAND6	cg08562141	0.131	0.085	4.26 × 10^−16^	1.12 × 10^−11^
ARHGEF7	cg07959338	0.129	0.085	4.41 × 10^−16^	1.12 × 10^−11^
UBXN8	cg08837587	0.135	0.093	4.73 × 10^−16^	1.12 × 10^−11^
USF1	cg13382707	0.107	0.070	5.04 × 10^−16^	1.12 × 10^−11^
USP12	cg01517521	0.104	0.065	5.79 × 10^−16^	1.20 × 10^−11^

**Table 3:** Methylation values of known tumor suppressor genes in the WTC-exposed (WTC EHC) cancer-free subjects compared to the unexposed cancer-free subjects (NYUWHS).

Gene	Probe ID	WTC EHC Methylation Value, Mean	NYUWHS Methylation Value, Mean	Methylation Status *	Regulatory Feature
ATM	cg18457775	0.056	0.042	Higher	Promoter
BCL11B	cg03498048	0.114	0.191	Lower	Promoter
CDK6	cg12548629	0.084	0.057	Higher	Promoter
CDKN2C	cg07013994	0.054	0.042	Higher	Promoter
CLYD	cg06458795	0.051	0.039	Higher	Promoter
IDH1	cg18755114	0.103	0.071	Higher	Promoter
JAK2	cg03693943	0.078	0.057	Higher	Promoter
NOTCH1	cg04271687	0.059	0.041	Higher	Promoter
NF1	cg25204988	0.052	0.041	Higher	Promoter
NUP98	cg20457962 cg01954337	0.154 0.192	0.121 0.144	Higher Higher	Promoter Promoter
PALB2	cg15645140 cg07627390	0.052 0.099	0.037 0.067	Higher Higher	Promoter Promoter
PTEN	cg18665732 cg16687447	0.054 0.053	0.038 0.038	Higher Higher	Promoter Promoter
SOCS1	cg11973052	0.047	0.035	Higher	Promoter
TCF3	cg24680852	0.068	0.045	Higher	Promoter
TSC1	cg11295002	0.092	0.066	Higher	Promoter
TSC2	cg02263479	0.051	0.038	Higher	Promoter
WRN	cg03410815	0.115	0.075	Higher	Promoter
CDH11	cg09631415	0.505	0.601	Lower	NA
MEN1	cg15893070	0.203	0.160	Higher	NA
RUNX1	cg01337293	0.096	0.069	Higher	NA
SUFU	cg01357317	0.858	0.804	Higher	NA
TNFAIP3	cg14527802	0.055	0.042	Higher	NA
SDHD	cg11542469	0.049	0.037	Higher	Non-Gene Associated
MLH1	cg25837710 cg06590608	0.050 0.039	0.036 0.067	Higher Lower	Unclassified Unclassified

* Methylation status refers to mean methylation value of the WTC exposed subjects relative to mean methylation value of unexposed subjects.

## Supplementary Materials

The following are available online at https://www.mdpi.com/1660-4601/17/15/5493/s1, Table S1: Methylation values of known oncogenes in the WTC-exposed (WTC EHC) cancer-free subjects compared to the unexposed cancer-free subjects (NYUWHS).
