# Supplementary material for: Genome-Wide DNA Methylation Profiles in Community Members Exposed to the World Trade Center Disaster
Source: Int J Environ Res Public Health. 2020 Jul 30;17(15):5493. doi: 10.3390/ijerph17155493 (PMC7432006; doi:10.3390/ijerph17155493)
Supplement: Supplementary file 1 [file ijerph-17-05493-s001.pdf]

Supplemental Table S1. Methylation values of known oncogenes in the WTC-exposed (WTC EHC) cancer-free subjects compared to the unexposed cancer-free subjects (NYUWHS)

Oncogenes					
**Gene**	**Probe ID**	**WTC EHC methylation value, mean**	**NYUWHS methylation value, mean**	**Methylation Status***	**Regulatory Feature**
**BCL3**	cg16632661	0.101	0.068	Higher	NA
**BCL6**	cg12110155	0.844	0.801	Higher	Promoter
	cg24771349	0.047	0.036	Higher	NA
**BRAF**	cg14094063	0.111	0.073	Higher	Promoter
	cg23066879	0.038	0.032	Higher	Promoter
**CARD11**	cg26937500	0.126	0.089	Higher	Promoter
**CBLB**	cg00522034	0.055	0.038	Higher	Promoter
	cg05404618	0.050	0.035	Higher	Promoter
**CCND2**	cg24626079	0.052	0.039	Higher	NA
**CCND3**	cg10142984	0.094	0.061	Higher	Promoter
**CTNNB1**	cg00052422	0.052	0.040	Higher	NA
	cg19155557	0.284	0.208	Higher	Promoter
**DDIT3**	cg17660561	0.053	0.039	Higher	Promoter
**DEX**	cg21091119	0.068	0.048	Higher	Promoter
	cg07636757	0.064	0.046	Higher	NA
**ELK4**	cg03126043	0.057	0.043	Higher	Promoter
**ERBB2**	cg12648523	0.860	0.805	Higher	NA
	cg27300230	0.057	0.044	Higher	Promoter
**ETV4**	cg13357178	0.072	0.055	Higher	NA
**ESWR1**	cg23827657	0.140	0.100	Higher	NA
	cg20611294	0.063	0.045	Higher	Promoter
	cg20359783	0.059	0.043	Higher	Promoter
	cg02226779	0.057	0.042	Higher	Promoter
**FGFR1OP**	cg13528713	0.062	0.044	Higher	Promoter
**GOPC**	cg06104144	0.048	0.034	Higher	Promoter
**HMGA1**	cg22481253	0.156	0.107	Higher	Promoter
**IRF2**	cg09764206	0.046	0.033	Higher	Promoter
**JUN**	cg20787340	0.065	0.047	Higher	Promoter
**KRAS**	cg08578305	0.074	0.047	Higher	Promoter
	cg13085893	0.077	0.052	Higher	Promoter
	cg13532571	0.084	0.057	Higher	Promoter
**MDM2**	cg05953180	0.072	0.054	Higher	Promoter
**MYCN**	cg17519750	0.094	0.070	Higher	NA
**NFKB2**	cg01013447	0.167	0.130	Higher	NA
**NTRK1**	cg09188405	0.821	0.771	Higher	NA
**NUP214**	cg25179280	0.052	0.039	Higher	Promoter
**PLAG1**	cg17286208	0.077	0.053	Higher	Promoter
**REL**	cg01438892	0.155	0.113	Higher	Promoter
**SS18**	cg12456090	0.069	0.048	Higher	Promoter
**TFG**	cg16052390	0.075	0.055	Higher	Unclassified
	cg11094012	0.153	0.116	Higher	Unclassified

*Methylation status refers to mean methylation value of the WTC exposed subjects (WTC EHC) relative to mean methylation value of unexposed subjects (NYUWHS).
